# Supplementary material for: Reproducibility analysis of automated deep learning based localisation of mandibular canals on a temporal CBCT dataset
Source: Sci Rep. 2023 Aug 29;13:14159. doi: 10.1038/s41598-023-40516-8 (PMC10465591; doi:10.1038/s41598-023-40516-8)
Supplement: Supplementary file 1 — Supplementary Information. [file 41598_2023_40516_MOESM1_ESM.pdf]

Supplementary Information for reproducibility analysis of automated deep learning based localisation of mandibular canals on a temporal CBCT dataset

Jorma Järnstedt[1,2], Jaakko Sahlsten[3], Joel Jaskari[3], Kimmo Kaski[3,6,*], Helena Mehtonen[1], Ari Hietanen[4], Osku Sundqvist[4], Vesa Varjonen[4], Vesa Mattila[4], Sangsom Prapayasatok[5], and Sakarat Nalampang[5]

[1]Medical Imaging Centre, Department of Radiology Tampere University Hospital, Teiskontie 35, 33520 Tampere, Finland
[2]The Graduate School, Chiang Mai University, 239 Huaykaew Road, Suthep, Meuang, Chiang Mai, Thailand
[3]Aalto University School of Science, Maarintie 8, 02150 Aalto, Finland
[4]Planmeca Oy, Asentajankatu 6, 00880 Helsinki, Finland
[5]Division of Oral and Maxillofacial Radiology, Faculty of Dentistry, Chiang Mai University, Suthep Rd., T. Suthep, A. Muang, Chiang Mai, Thailand
[6]Alan Turing Institute, British Library, 96 Euston Rd, London NW1 2DB, UK
[*]Corresponding author, kimmo.kaski@aalto.fi

Supplementary methods

This section includes more detailed description of the patient data and the derivation of the Bayesian statistical method of our reproduciblity analysis.

Patient data

The number of CBCT scans with different devices and voxel spacings are presented in Table S1.

Manufacturer	Device	Dose	Voxel spacing	Scans
KaVo	KAVO OP 3D Pro	HR	0.38	7 (4.2%)
Planmeca	VISO G7	HR	0.15	1 (0.6%)
			0.20	17 (10.3%)
			0.30	8 (4.8%)
			0.45	1 (0.6%)
		LD	0.30	12 (7.3%)
		SR	0.20	3 (1.8%)
			0.30	3 (1.8%)
			0.40	7 (4.2%)
			0.45	1 (0.6%)
		ULD	0.30	8 (4.8%)
			0.40	5 (3.0%)
SOREDEX	Scanora 3Dx	HR	0.20	73 (44.2%)
		SR	0.20	1 (0.6%)
			0.30	17 (10.3%)
			0.50	1 (0.6%)

Table S1. Distribution of scanners, doses, and voxel spacings for all the 165 CBCT scans utilised in this study.

The Bayesian method for reproducibility-analysis of ordinal data

The reproducibility results for Likert analysis utilise the Bayesian repeatability and reproducibility method, described in Culp et al.[1]. In Bayesian analysis, at least some of the parameters of the model are thought to be random and distributed according to a

prior distribution, and their posterior distribution is inferred given the likelihood of the data with the Bayes' theorem:

$$p(\theta \mid D) = \frac{p(D \mid \theta)p(\theta)}{p(D)}. \tag{1}$$

The model in Culp et al.[1] is based on the De Mast-Van Wieringen model[2]. It assumes that the subject-specific random effects x_i are distributed as standard normal, i.e. that $x_i \sim \mathcal{N}(0,1)$. Each grader j is determined by their parameters α_j and δ_j. The parameter α_j can be thought to represent the confidence or discrimination ability of a grader, such that a higher value of α_j indicates a higher ability to separate the Likert grades. The elements of the grader specific parameter vector $\delta_j = [\delta_{j,1}, \ldots, \delta_{j,(H-1)}]$ are ordered cut-point or threshold parameters, which determine the boundaries of the outcomes. The elements have the following property $\delta_{jn} < \delta_{jm}$, $\forall n < m$, and if the random effect x_i falls between the interval: $\delta_{j(h-1)} < x_i < \delta_{jh}$, the most likely Likert grade the grader j will give is h according to the model.

The likelihood model and the priors, as given by the study, are:

$$p(y_{i,j} = h \mid x_i, \alpha_j, \delta_j) = \frac{\exp\left(\sum_{m=1}^{h-1} \alpha_j (x_i - \delta_{j,m})\right)}{\sum_{n=1}^{H} \exp\left(\sum_{m=1}^{n-1} \alpha_j (x_i - \delta_{j,m})\right)}, \tag{2}$$

$$p(x_i) = \mathcal{N}(x \mid 0,1), \tag{3}$$

$$p(\alpha_j) \propto \alpha_j^{-1} \exp(-\frac{1}{2\alpha}), \tag{4}$$

$$\delta_{jm} = \Phi^{-1}\left(\sum_{n=1}^{m} \pi_{j,n}\right), \tag{5}$$

$$p(\pi_j) = \text{Dirichlet}(\pi_j \mid \left[\frac{1}{2}, \ldots, \frac{1}{2}\right]), \tag{6}$$

where $y_{i,j}$ is the Likert grade subject i received from grader j. Similar to the original work, multiple grades are modelled simultaneously using the multinomial distribution with the repetitions treated as count data: $f(c_{i,j,1}, \ldots, c_{i,j,5} \mid n_i, p_{i,j,1}, \ldots, p_{i,j,5})$, where $c_{i,j,h}$ is the number of grade h:s that the grader j gave to the subject i, n_i is the total number of repeats for the subject i, and $p_{i,j,h}$ is given by (2).

In practice, we utilize the BUGS program code provided in the Appendix of the work[1], which we converted to a Stan[3] program. It samples parameters from the posterior distribution, given in (1), with $\theta = [\alpha_1, \alpha_2, \alpha_3, \delta_1, \delta_2, \delta_3, x_1, \ldots, x_N]$ and $D = [c_{1,1,1}, c_{1,1,2}, \ldots, c_{N,3,4}, c_{N,3,5}]$. We used the default number of posterior samples, burn-in samples, and Markov Chain Monte Carlo chains of Stan. The posterior samples are used to compute the repeatability measure (RM), proposed in Culp et al.[1] (Equation (21) in the work), the posterior mean of which is computed for each expert j:

$$RM_j^{(m)} = \frac{1}{N} \sum_{i=1}^{N} \sum_{h=1}^{5} p(y_{ij} = h \mid x_i, \alpha_j^{(m)}, \delta_j{}^{(m)})^2, \tag{7}$$

where the superscript (m) indices the posterior sample. Our main results are the posterior average of the RM (i.e. $RM_j = 1/M \sum_m RM_j^{(m)}$), and we also report the average RM of all the experts (i.e. $(RM_1 + RM_2 + RM_3)/3$).

Supplementary results

This section presents supplementary results for both the quantitative and qualitative analyses.

Quantitative results

For quantitative results, the immediate post-operative (IPO) scans had an overall performance with median (IQR) SMCD of 1.085 (1.867) mm for 40 valid canals, median (IQR) ASSD of 0.618 (2.55) mm for 40 valid canals, and mean (standard deviation) DSC of 0.352 (0.202) for all 46 canals. In addition, the mean (standard deviation) for sensitivity and false positive rate (FPR) for IPO canals were 0.462 (0.336) and $2.8 \times 10^{-5} (1.7 \times 10^{-5})$, respectively, and for valid canals the median (IQR) Hausdorff distance (HD) was 8.911 (32.239) mm. The overall results are shown in Figure S1 and patient-wise results are show in Figure S4. Within subject repeatability results, including the IPO scans, are shown in Table S2. Within subject repeatability results when grouping by gender, age, and device are reported in Table S3. All reported error types are shown in Table S6. For all the canals, the mean (standard deviation) sensitivity and FPR were 0.655 (0.148) and $3.5 \times 10^{-5} (1.3 \times 10^{-5})$, respectively, and for all the valid canals, the median (IQR) HD was 2.298 (1.680) mm. As stated in the main body; the worse performance of

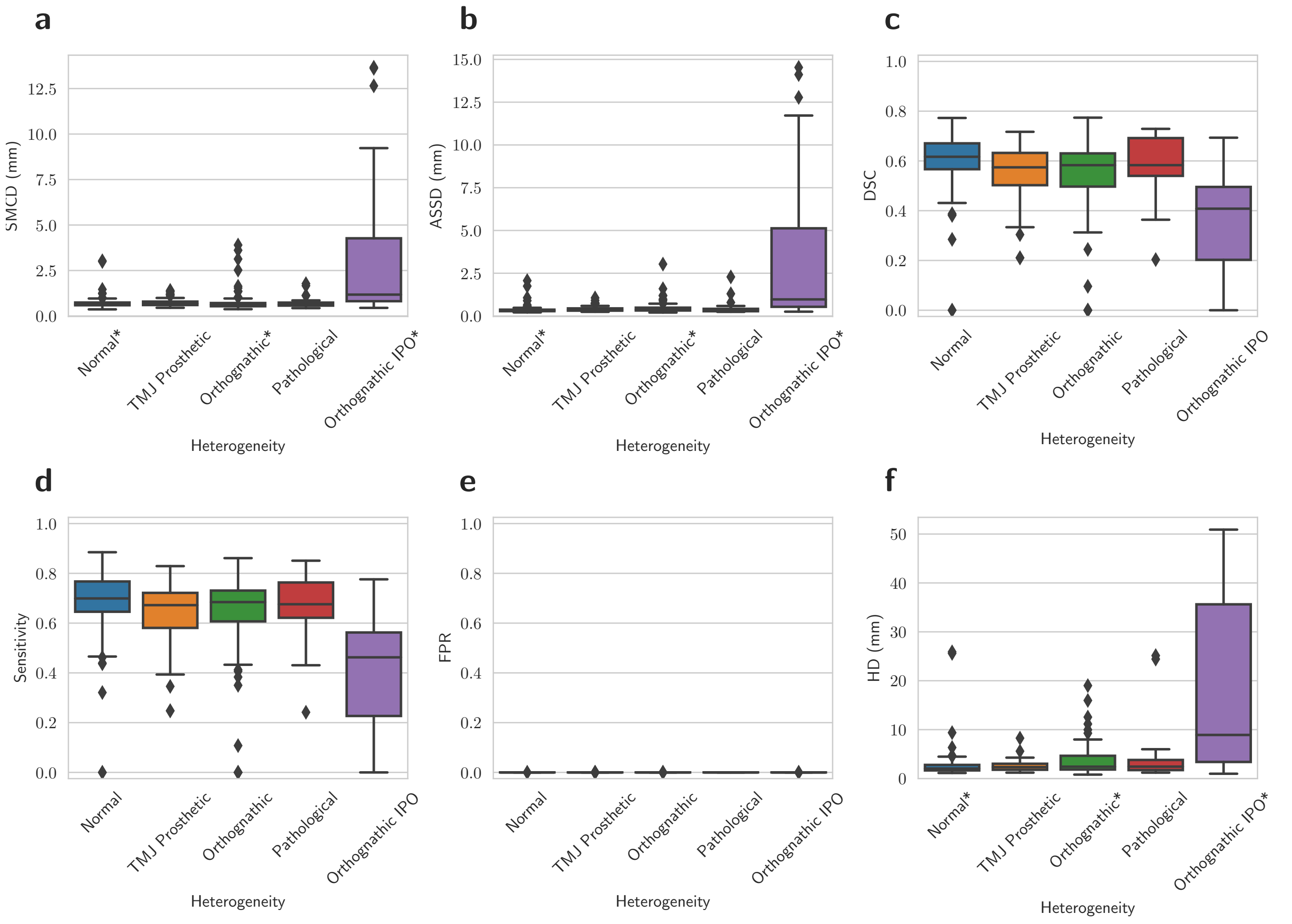

Figure S1. Overall results including the immediate post-operative (IPO) scans. a. Symmetric mean curve distance (mm), b. Average symmetric surface distance (mm), c. Dice similarity coefficient, d. Sensitivity, e. False positive rate, f. Hausdorff distance. $^{*)}$two, four, and five missing canals are omitted for Normal, Orthognathic, and Orthognathic IPO groups, respectively.

the DLS on the IPO scans is expected, as the visibility of the canals is extremely poor in them[4] and there are changes to the path of the mandibular canal due to the surgery.

In addition, we analyzed the quantitative performance when grouping the canals based on the device and dosage. The median SMCD values were 0.636, 0.660, and 0.643 mm for KaVO OP 3D Pro, VISO G7, and Scanora 3Dx, respectively. The median ASSD values were 0.342, 0.386, and 0.336 mm for KaVO OP 3D Pro, VISO G7, and Scanora 3Dx, respectively. The median DSC values were 0.568, 0.516, and 0.603 mm for KaVO OP 3D Pro, VISO G7, and Scanora 3Dx, respectively. As for grouping by dose, the median SMCD values were 0.640, 0.630, 0.672, 0.839 mm for HR, SR, LD, and ULD, respectively. The median ASSD values were 0.333, 0.354, 0.451, 0.582 mm for HR, SR, LD, and ULD, respectively. The mean DSC values were 0.601, 0.559, 0.474, 0.410 for HR, SR, LD, and ULD, respectively. Full results for the device and dose grouping are shown in Figure S3.

In addition, the overall performance of the DLS was evaluated when grouping canals based on 10 year interval age bins and gender. With females the median SMCD value was 0.640, 0.700, 0.631, 0.680, 0.682, 0.878 mm for ≤ 30, $31-40$, $41-50$, $51-60$, $61-70$, >70 ages, respectively. With males the median SMCD value was 0.663, 0.640, 0.613, 0.613, 0.646, 0.630 mm for ≤ 30, $31-40$, $41-50$, $51-60$, $61-70$, >70 ages, respectively. With females the median ASSD value was 0.346, 0.478, 0.361, 0.351, 0.356, 0.473 mm for ≤ 30, $31-40$, $41-50$, $51-60$, $61-70$, >70 ages, respectively. With males the median ASSD value was 0.342, 0.341, 0.412, 0.340, 0.367, 0.308 mm for ≤ 30, $31-40$, $41-50$, $51-60$, $61-70$, >70 ages, respectively. With females the mean DSC value was 0.593, 0.500, 0.569, 0.552, 0.545, 0.461 for ≤ 30, $31-40$, $41-50$, $51-60$, $61-70$, >70 ages, respectively. With males the mean DSC value was 0.560, 0.600, 0.504, 0.525, 0.576, 0.659 for ≤ 30, $31-40$, $41-50$, $51-60$, $61-70$, >70 ages, respectively. Full results are shown in Figure S4.

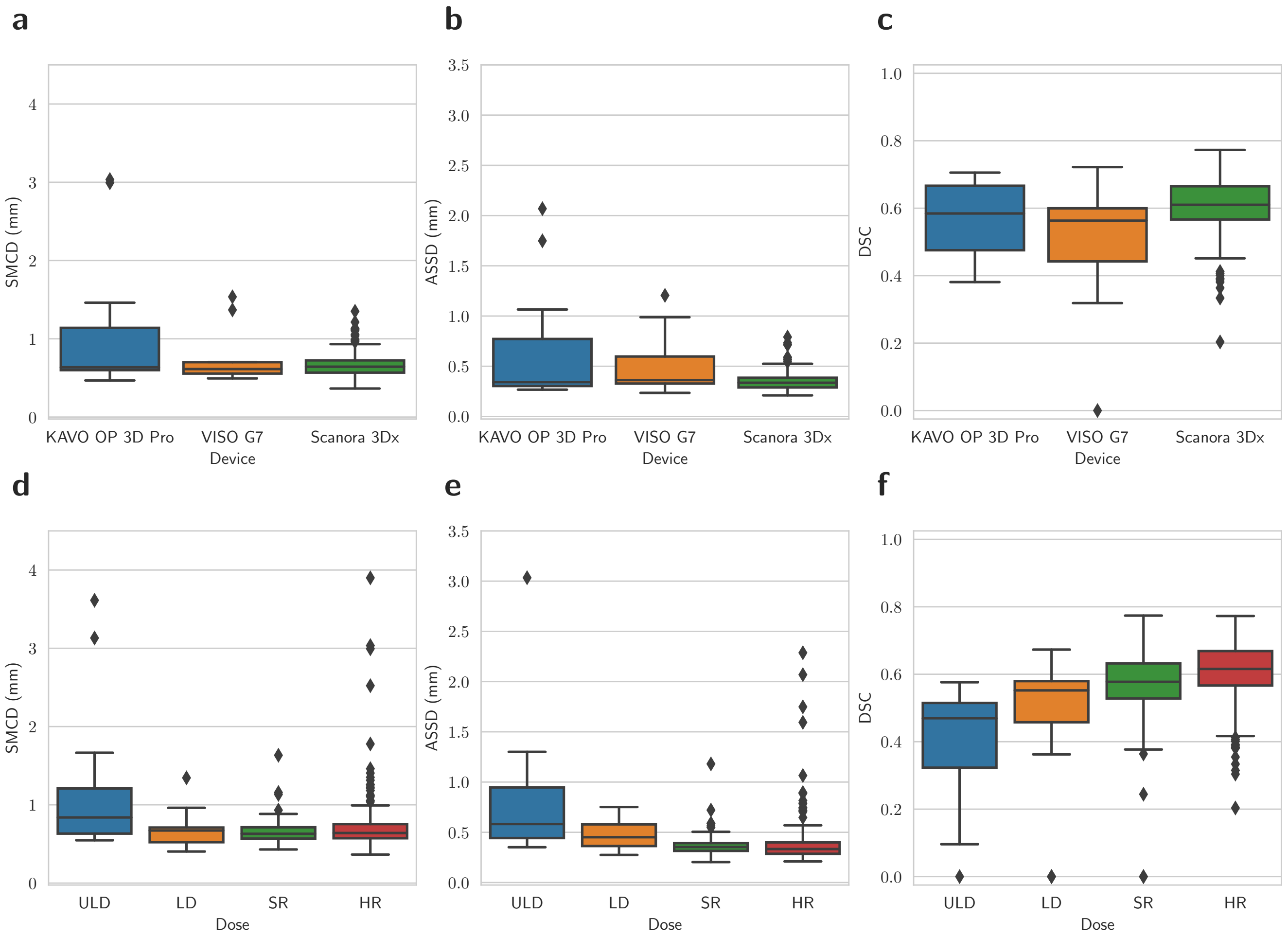

Figure S2. Quantitative performance comparison of the deep learning system between the different device groups shown in subfigures without the immediate post-operative scans evaluated with a. symmetric mean curve distance, b. average symmetric surface distance, and c. Dice similarity coefficient. Comparison of performance between dose groups shown in subfigures without the immediate post-operative scans evaluated with d. symmetric mean curve distance, e. average symmetric surface distance, f. Dice similarity coefficient.

Qualitative results

We performed additional Bayesian reproducibility analysis on groups not considered in our main results. Results for the immediate post-operative scans are presented in Table S4, Figure S7, and Figure S11. It can be seen that the repeatability measures of both the Radiologist and DLS drop when including the IPO scans, in comparison to using non-IPO Orthognathic group scans. Additional reproducibility results, when grouping by gender, age, and device, are shown in Table S5. The Bayesian statistical analysis of Likert scores provides posterior samples of the repeatability measure, which is computed with Equation (7). Figure S5 illustrates the distributions of RMs for each heterogeneity group and the full dataset RM distributions are presented in Figure S6.

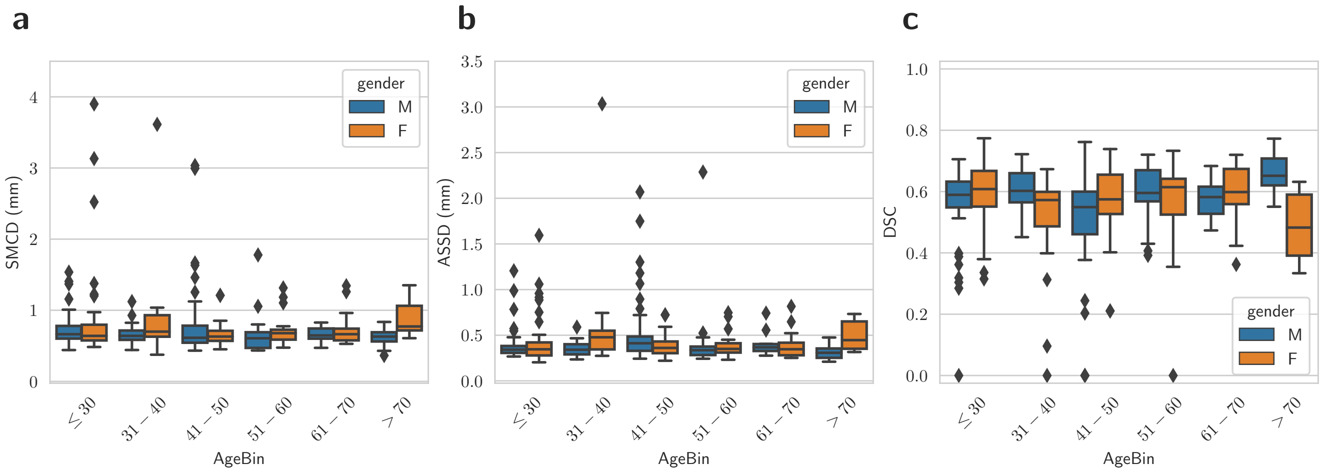

Figure S3. Comparison of quantitative performance of the deep learning system between the different age groups in 10 year interval bins. a. symmetric mean curve distance, b. average symmetric surface distance, c. Dice similarity coefficient.

Heterogeneity	n	N	K	Mean	wSD	RC	Range
Full dataset with IPO	134	345	{2, 3, 4, 5, 6}	0.962	1.419	3.931	[0.487, 7.088]
Full dataset	131	302	{2, 3, 4, 5}	0.761	0.350	0.969	[0.471, 3.014]
Orthognathic with IPO	43	127	{2, 3, 6}	1.430	2.240	6.204	[0.492, 7.088]
Orthognathic	40	84	{2, 4}	0.806	0.616	1.707	[0.471, 2.206]

Table S2. Within-subject mean and standard deviation (wSD), repeatability coefficient (RC), and the range of valid SMCD (mm) values with at least two temporal canals. Results evaluated for data with and without the immediate post-operative (IPO) scans. N = the total number of canals, n = the number of unique canals, and K = the set of temporal samples per canal.

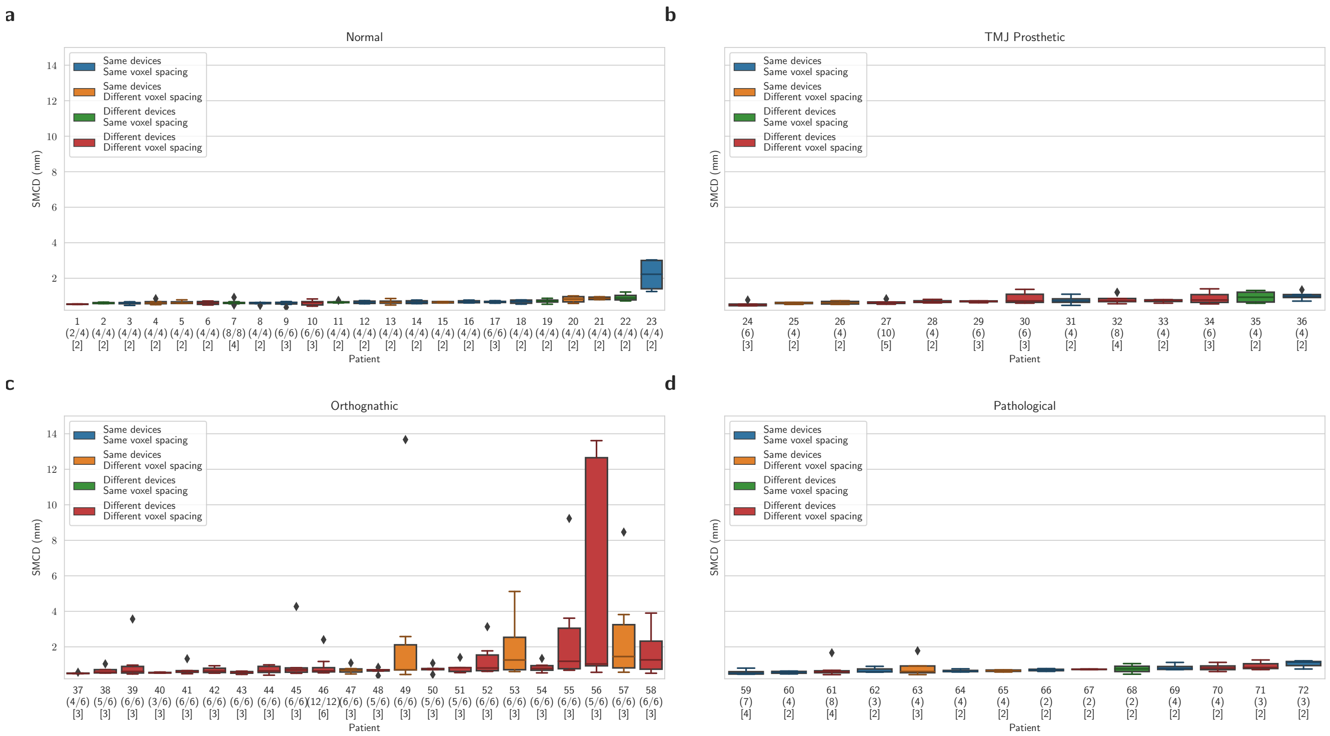

Figure S4. Patient-wise results for Orthognathic group including the immediate post-operative (IPO) scans. a. Normal, b. TMJ Prosthetic, c. Orthognathic, d. Pathological.

Gender/Age/Device	n	N	K	Mean	wSD	RC	Range
Full dataset	131	302	{2, 3, 4, 5}	0.761	0.350	0.969	[0.471, 3.014]
F	66	152	{2, 3, 4, 5}	0.793	0.452	1.252	[0.471, 2.206]
M	65	150	{2, 3, 4}	0.729	0.199	0.551	[0.494, 3.014]
≤ 50	87	204	{2, 3, 4, 5}	0.791	0.403	1.115	[0.471, 3.014]
> 50	44	98	{2, 3, 4}	0.703	0.190	0.527	[0.510, 1.172]
Female of age ≤ 50	41	100	{2, 3, 4, 5}	0.815	0.525	1.454	[0.471, 2.206]
Female of age > 50	25	52	{2, 3}	0.757	0.225	0.624	[0.558, 1.172]
Male of age ≤ 50	46	104	{2, 3, 4}	0.769	0.219	0.606	[0.494, 3.014]
Male of age > 50	19	46	{2, 3, 4}	0.631	0.147	0.407	[0.510, 0.757]
KaVo KAVO OP 3D Pro	6	12	{2}	1.111	0.095	0.264	[0.554, 3.014]
Planmeca VISO G7	28	59	{2, 3}	0.734	0.224	0.621	[0.504, 1.281]
SOREDEX Scanora 3Dx	50	115	{2, 3, 4}	0.684	0.132	0.366	[0.515, 1.172]

Table S3. Within-subject mean and standard deviation (wSD), repeatability coefficient (RC), and the range of valid SMCD (mm) values. The results are presented for the full dataset and for different demographic and devicegroups separately. N = the total number of canals, n = the number of unique canals, and K = the set of the number of canals present in the group.

Reproducibility	Heterogeneity	Radiologist Mean (95% CI)	DLS Mean (95% CI)
Average	Orthognathic	**0.945 (0.844, 1.000)**	0.815 (0.711, 0.907)
	Orthognathic IPO	**0.804 (0.586, 0.975)**	0.584 (0.459, 0.679)
Expert 1	Orthognathic	**0.995 (0.970, 1.000)**	0.866 (0.796, 0.917)
	Orthognathic IPO	**0.953 (0.907, 0.981)**	0.623 (0.551, 0.686)
Expert 2	Orthognathic	**0.947 (0.888, 0.982)**	0.793 (0.704, 0.861)
	Orthognathic IPO	**0.815 (0.746, 0.874)**	0.619 (0.548, 0.684)
Expert 3	Orthognathic	**0.893 (0.821, 0.944)**	0.786 (0.698, 0.856)
	Orthognathic IPO	**0.645 (0.562, 0.722)**	0.510 (0.440, 0.576)

Table S4. Reproducibility results, in terms of the repeatability measure, for the Likert scoring of the radiologist's annotations and the DLS outputs when using the Orthognathic IPO scans, in comparison to excluding them (Orthognathic).

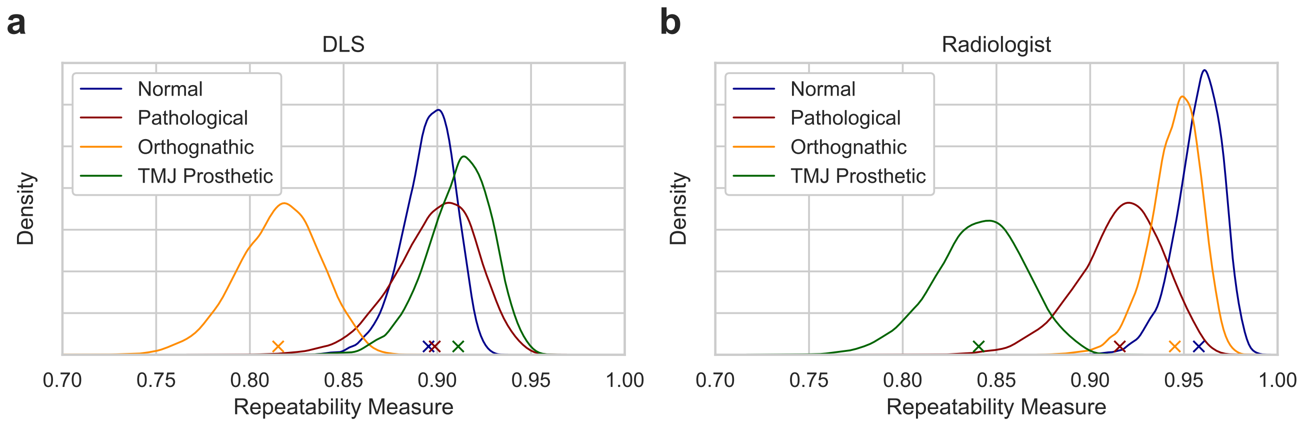

Figure S5. Distribution of repeatability measures for each heterogeneity group, a. DLS, b. Radiologist, the posterior mean value (main results) indicated with a cross symbol.

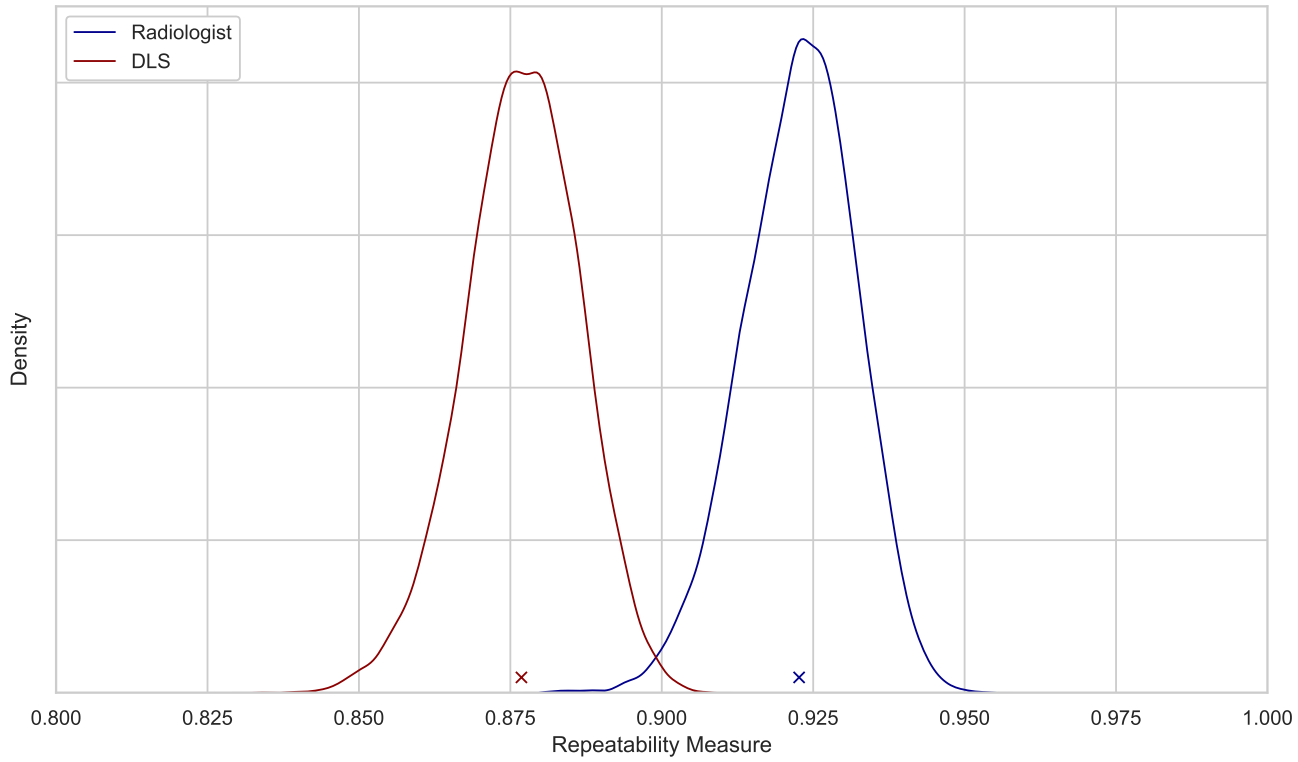

Figure S6. Distribution of repeatability measures when analysing the entire dataset without heterogeneity grouping, the posterior mean value indicated with a cross symbol.

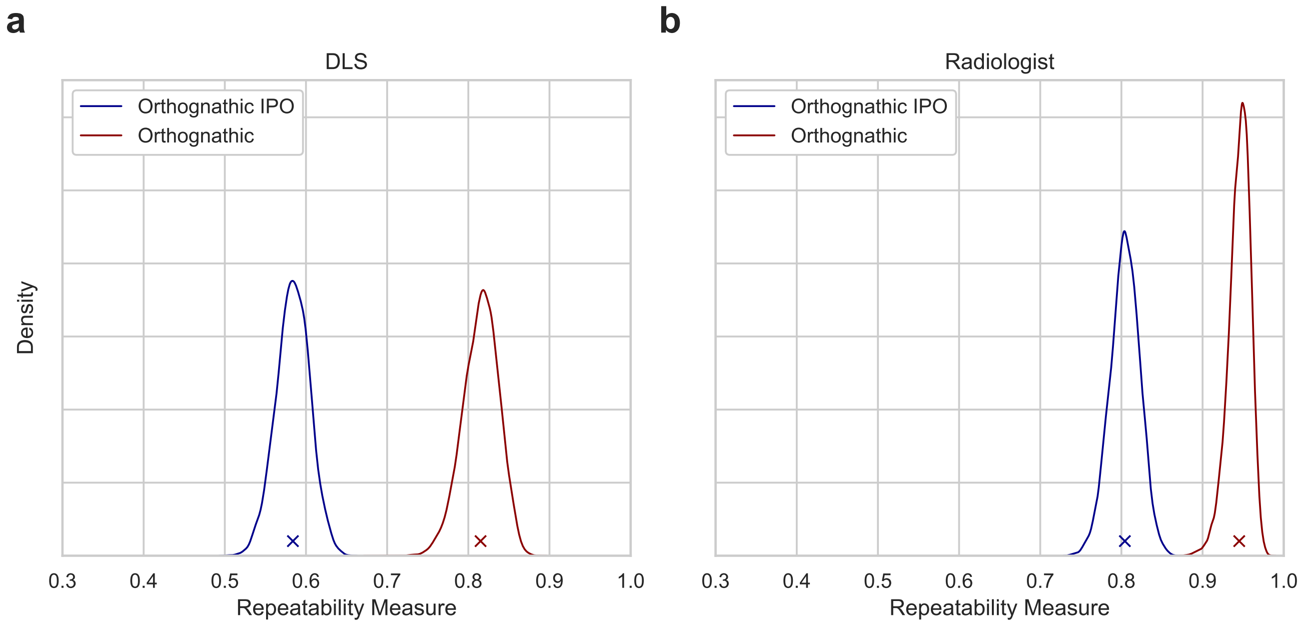

Figure S7. Distribution of repeatability measures when including the IPO scans (Orthognathic IPO) in comparison to excluding them (Orthognathic), a. DLS and b. Radiologist.

Grouping	Radiologist Mean (95% CI)	DLS Mean (95% CI)
Female	0.928 (0.878, 0.968)	0.861 (0.779, 0.925)
Male	0.921 (0.788, 0.997)	0.860 (0.784, 0.922)
≤ 50	0.918 (0.827,0.984)	0.835 (0.755,0.904)
> 50	0.938 (0.852,0.985)	0.911 (0.834,0.960)
Female of Age ≤ 50	0.921 (0.853,0.970)	0.839 (0.752,0.912)
Female of Age > 50	0.949 (0.863,0.990)	0.880 (0.740,0.956)
Male of Age ≤ 50	0.919 (0.764,1.000)	0.824 (0.715,0.905)
Male of Age > 50	0.929 (0.795,0.996)	0.961 (0.864,1.000)
SOREDEX Scanora 3Dx	0.949 (0.884, 0.992)	0.955 (0.900, 0.988)
Planmeca VISO G7	0.927 (0.820, 0.992)	0.890 (0.801, 0.977)
KaVo KAVO OP 3D Pro	0.876 (0.575, 1.000)	0.761 (0.461, 0.961)

Table S5. Additional qualitative reproducibility results, in terms of the repeatability measure, for the Likert scoring of the radiologist's annotation and the DLS output. Results shown when grouping by gender, two age groups, combination of the two aformentioned groups, as well as grouping by the device. CI denotes the Bayesian credibility interval.

Segmentation	Type	Heterogeneity	Percentage (%)	Count
Annotation	Major parts missing	TMJ Prosthetic	0.5	1
	Short at mandibular foramen	Orthognathic	0.4	1
		Pathological	0.5	1
		TMJ Prosthetic	2.9	6
	Slightly off centre	Normal	1	3
		Orthognathic	0.4	1
		Pathological	1	2
		TMJ Prosthetic	1.4	3
DLS	Fully missing	Normal	2	6
		Orthognathic	4	11
	Major parts missing	Orthognathic	0.4	1
		TMJ Prosthetic	1	2
	Short at mandibular foramen	Orthognathic	0.7	2
		Pathological	1.5	3
		TMJ Prosthetic	1.4	3
	Short at mental foramen	Normal	2	6
		Orthognathic	2.2	6
		Pathological	2	4
	Slightly off centre	Normal	0.7	2
		Orthognathic	0.4	1
		Pathological	0.5	1
		TMJ Prosthetic	0.5	1

Table S6. Distribution of error types observed in the Radiologist's annotations and in the DLS outputs, grouped by heterogeneity, showing all the Experts' reported values (N=990).

Table S7. Mean, standard deviation (SD), and counts of all the Likert ratings (N_L for the Likert score L) grouped by the gender, age, device, and the marker (the radiologist or the DLS). Higher mean score of each category bolded.

Gender/Age/Device/Dose	Marking	Mean	SD	N_0	N_1	N_2	N_3	N_4
Full dataset	Radiologist	**3.94**	**0.27**	0	3	12	72	1821
	DLS	3.84	0.65	35	9	35	75	1753
F	Radiologist	**3.94**	**0.31**	0	3	8	36	907
	DLS	3.85	0.59	11	7	20	36	879
M	Radiologist	**3.95**	**0.23**	0	0	4	36	914
	DLS	3.82	0.70	24	2	15	39	874
≤ 30	Radiologist	**3.96**	**0.21**	0	0	2	18	532
	DLS	3.86	0.57	6	3	12	18	513
$31-40$	Radiologist	**3.94**	**0.26**	0	0	2	13	273
	DLS	3.82	0.65	5	0	8	17	257
$41-50$	Radiologist	**3.93**	**0.31**	0	1	4	19	420
	DLS	3.71	0.88	18	3	9	29	385
$51-60$	Radiologist	**3.98**	**0.15**	0	0	0	6	264
	DLS	3.87	0.64	6	1	2	3	258
$61-70$	Radiologist	3.93	0.29	0	0	2	9	181
	DLS	**3.96**	**0.25**	0	0	2	4	186
> 70	Radiologist	3.90	0.44	0	2	2	7	151
	DLS	**3.91**	**0.42**	0	2	2	4	154
KAVO OP 3D Pro	Radiologist	**3.88**	**0.39**	0	0	2	6	76
	DLS	3.60	0.70	0	0	10	14	60
VISO G7	Radiologist	**3.94**	**0.28**	0	1	7	25	723
	DLS	3.69	0.94	35	7	22	29	662
Scanora 3Dx	Radiologist	3.95	0.25	0	2	3	41	1022
	DLS	**3.96**	**0.24**	0	2	3	32	1031
ULD	Radiologist	**3.88**	**0.38**	0	0	3	13	140
	DLS	3.58	0.95	5	2	15	9	124
LD	Radiologist	**3.95**	**0.27**	0	0	2	3	139
	DLS	3.44	1.34	18	0	2	5	119
SR	Radiologist	**3.97**	**0.18**	0	0	0	13	383
	DLS	3.84	0.71	12	0	1	15	368
HR	Radiologist	**3.95**	**0.28**	0	3	7	43	1159
	DLS	3.92	0.37	0	7	17	46	1142

Additional results

Correlation between quantitative and qualitative results is shown visually in Figure S8 with a scatter plot between the median Likert rating and SMCD (mm), ASSD (mm), and DSC.

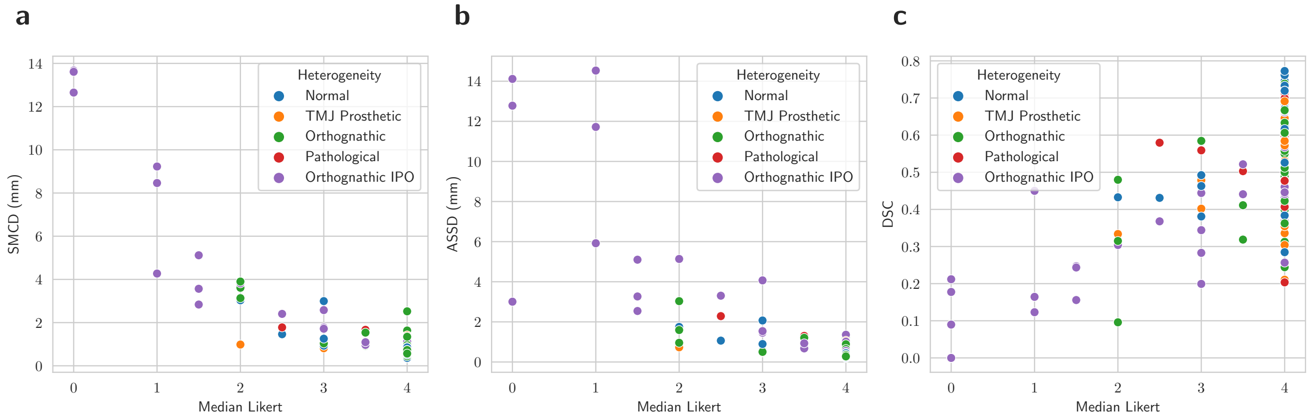

Figure S8. Scatterplot of median Likert compared to a. SMCD (mm) from valid canals, to b. Average symmetric surface distance (mm), and to c. Dice similarity coefficient from all canals.

Visual illustrations of the per canal posterior distribution of repeatability measures (i.e. when the average in Equation (7) is not computed) are shown in Figures S9, S10, and S11, for canals in each heterogeneity group, canals of the full dataset, and canals in the Orthognathic / Orthognathic IPO groups.

References

1. Culp, S. L., Ryan, K. J., Chen, J. & Hamada, M. S. Analysis of repeatability and reproducibility studies with ordinal measurements. *Technometrics* **60**, 545–556, DOI: 10.1080/00401706.2018.1429317 (2018).
2. de Mast, J. & van Wieringen, W. N. Modeling and evaluating repeatability and reproducibility of ordinal classifications. *Technometrics* **52**, 94–106, DOI: 10.1198/TECH.2009.08052 (2010).
3. Stan Development Team. Stan modeling language users guide and reference manual (2022). Version 2.30. https://mc-stan.org.
4. Politis, C. *et al.* Visibility of mandibular canal on panoramic radiograph after bilateral sagittal split osteotomy (BSSO). *Surg. Radiol. Anat.* **35**, 233–240 (2013).

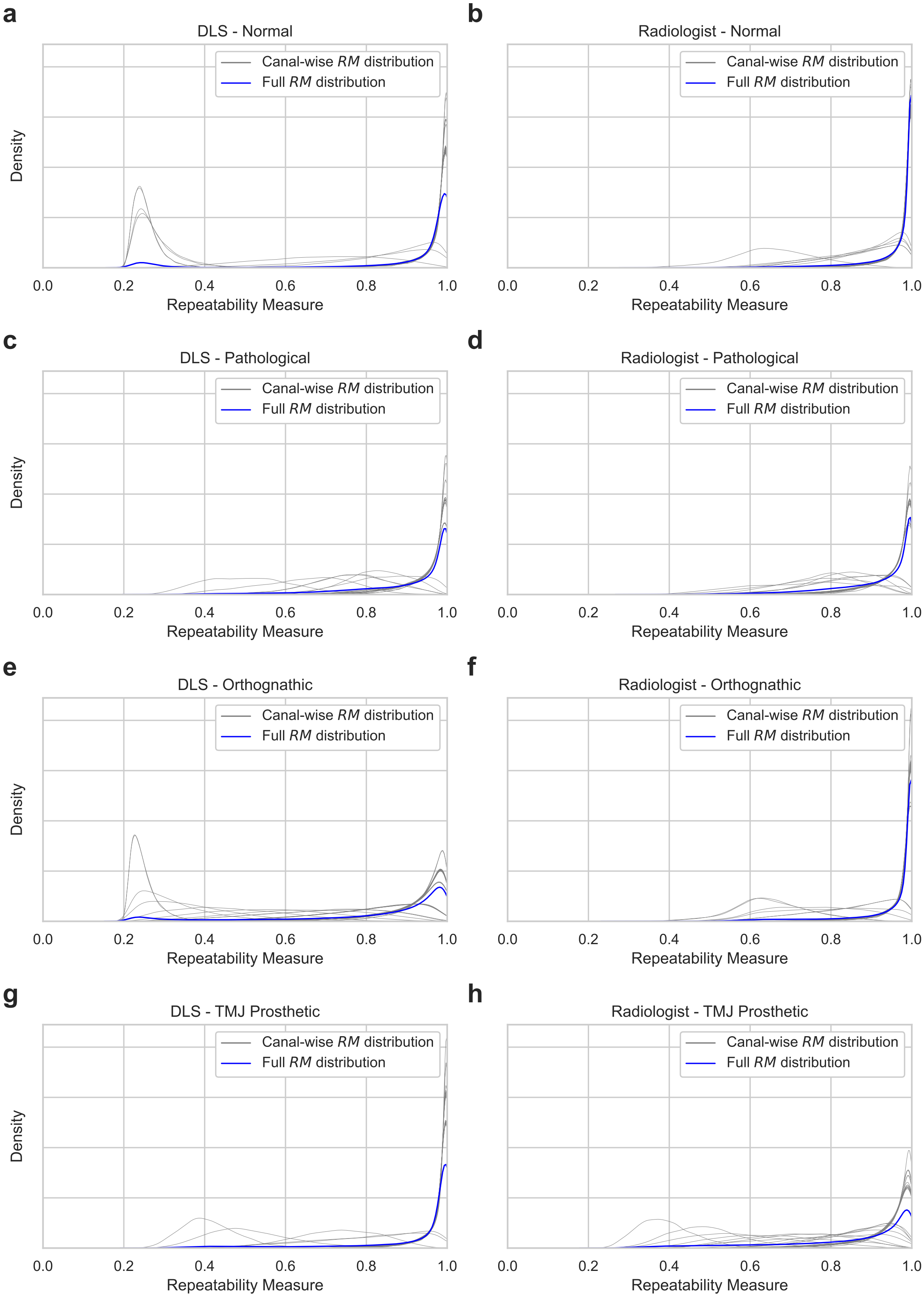

Figure S9. Distribution of repeatability measures per canal with gray lines indicating the distribution for each canal and blue lines the average density for each level of repeatability measure, first column (a., c., e., and g.) for DLS, second column (b., d., f., and h.) for Radiologist, first to last row for Normal, Pathological, Orthognatic, and TMJ Prosthetic groups, respectively.

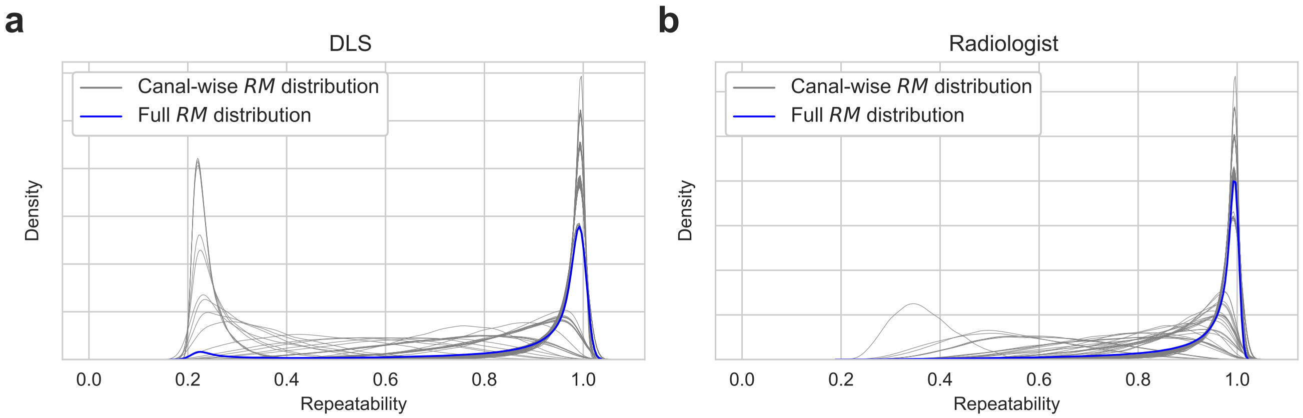

Figure S10. Distribution of repeatability measures per canal when analysing the entire dataset without heterogeneity grouping, gray lines indicate the distribution per canal and blue lines the average density for each level of repeatability measure.

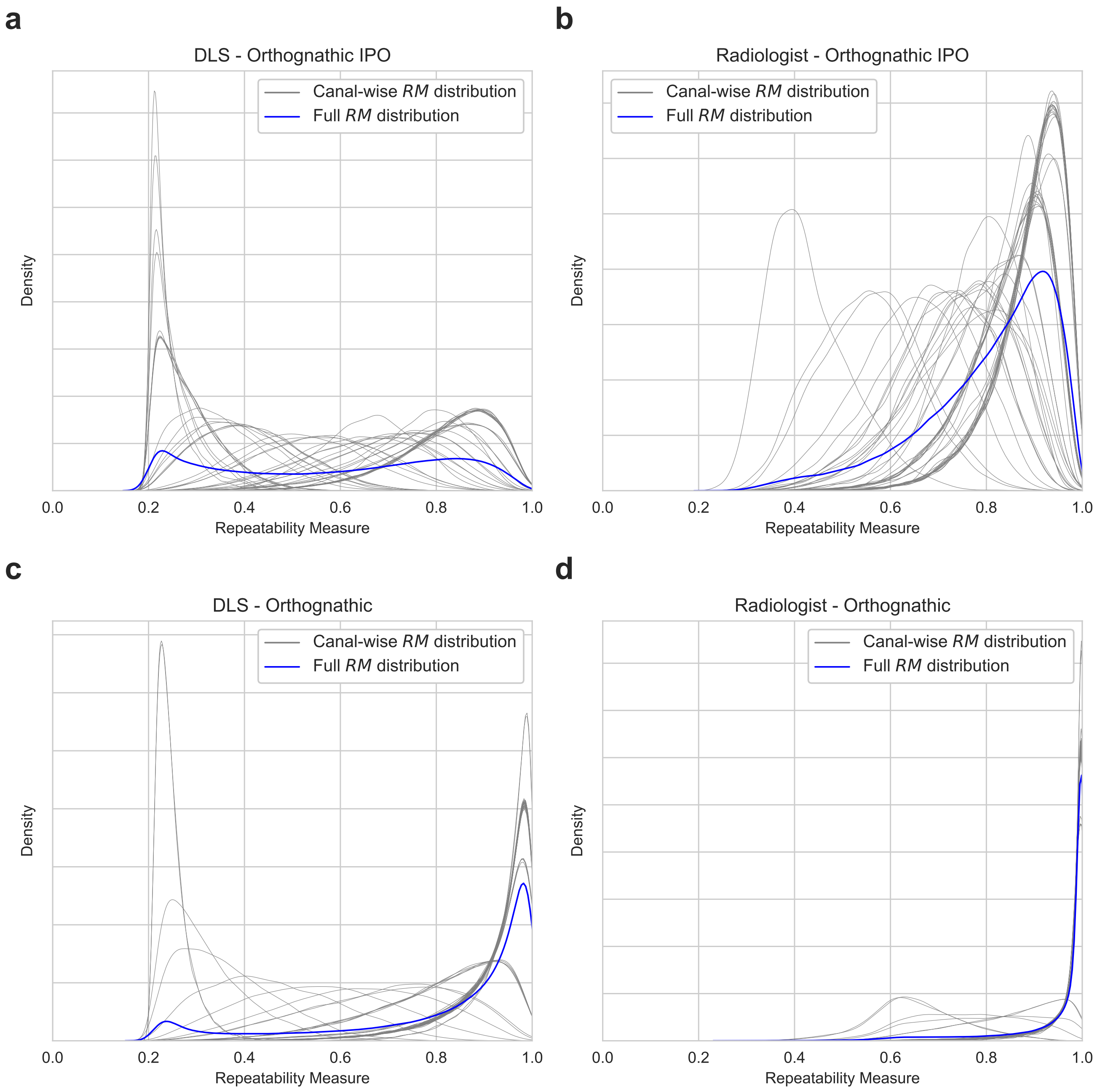

Figure S11. Distribution of repeatability measures per canal when including the IPO scans (Orthognathic IPO, a. and b.) in comparison to excluding them (Orthognathic, c. and d.), a. and c. DLS, and b. and d. Radiologist, gray lines indicate the distribution for each canal and blue lines the average density for each level of repeatability measure.
